# Phenylalanine Alleviates Postharvest Chilling Injury of Plum Fruit by Modulating Antioxidant System and Enhancing the Accumulation of Phenolic Compounds

**DOI:** 10.17113/ftb.58.04.20.6717

**Published:** 2020-12

**Authors:** Ommol Banin Sogvar, Vali Rabiei, Farhang Razavi, Gholamreza Gohari

**Affiliations:** 1 Department of Horticulture, Faculty of Agriculture, University of Zanjan, University Blvd., 45371-38791, Zanjan, Iran; 2 Department of Horticulture, Faculty of Agriculture, University of Maragheh, Daneshgah Blvd., Madar Square, 83111-55181, Maragheh, East Azarbaijan, Iran

**Keywords:** antioxidant capacity, chilling injury, phenylalanine, plum fruit, phenylalanine ammonia lyase (PAL)

## Abstract

**Research background:**

Low temperature storage causes chilling injury in plum (*Prunus domestica* L.) fruits. Consequently, any treatments with beneficial effects against these symptoms would achieve attention. For this purpose, phenylalanine treatments were applied on ‘Stanley’ plum fruits. The main purpose of the present study is to investigate the influence of the exogenous application of phenylalanine on fruit quality, chilling tolerance, and antioxidant capacity of ‘Stanley’ plums during cold storage.

**Experimental approach:**

Phenylalanine at different concentrations was applied on ‘Stanley’ plums. Following phenylalanine application, plums were cold stored. Chilling injury, antioxidant capacity, electrolyte leakage, malondialdehyde, proline and internal contents of anthocyanin, flavonoids, phenols, ascorbic acid and some antioxidant enzymes were assessed.

**Results and conclusions:**

Phenylalanine treatment signiﬁcantly alleviated chilling injury in plum fruits by enhancing antioxidant capacity and increasing the activity of phenylalanine ammonia lyase enzyme (PAL). Phenylalanine-treated fruits had higher mass fractions of ascorbic acid, anthocyanins, flavonoids and phenols, as well as a higher total antioxidant activity than the control fruits during low temperature storage. Phenylalanine at 7.5 mM was the most effective treatment in enhancing the activity of PAL, the accumulation of phenolic compounds and in reducing the severity of chilling injury. Treatments delayed mass loss and maintained fruit firmness. In addition, the application of 7.5 mM phenylalanine improved the activities of antioxidant enzymes (superoxide dismutase, catalase and ascorbate peroxidase), decreased the accumulation of hydrogen peroxide, and increased the endogenous content of proline. Moreover, phenylalanine maintained membrane integrity, manifested by a reduced electrolyte leakage and malondialdehyde accumulation.

**Novelty and scientific contribution:**

In the current study, chilling injury had a positive correlation with the activities of PAL and antioxidant enzymes. However, negative correlations were observed between the chilling injury and ascorbic acid mass fraction, and antioxidant capacity. Considering the results, phenylalanine treatment could be an encouraging approach to alleviate the severity of chilling injury and thus preserve nutritional quality of plums during low temperature storage.

## INTRODUCTION

Plum (*Prunus domestica* L.), a temperate fruit tree, is mostly cultivated for its fresh fruits around the world. Plums are considered excellent sources of fibre, carbohydrates, organic acids, potassium, calcium, vitamins C and E, polyphenols, carotenoids, flavonoids and anthocyanins, all of which are essential/beneficial for human health (*1*). The rapid fruit softening under ambient conditions is one of the most important challenges in the production of plum fruits. This is the main reason for the decrease of fruit quality during storage, transport and marketing. Therefore, low-temperature treatment is an efficient method for the reduction of the postharvest quality loss. However, plum fruits are highly susceptible to low temperatures, and the chilling injury symptoms show up as flesh browning, translucency, bleeding, and abnormal ripening when the fruits are kept at 0 or 5 °C (*2*). Thus, with the development of chilling symptoms, storage life is limited. However, cultivar, storage temperature, maturity stage and cultivation conditions affect the rate of chilling injury development in plums (*3*). Candan *et al*. (*4*) reported that the severity of chilling injury in ‘Larry Ann’ plums was high during cold storage.

Chilling stress triggers the accumulation of reactive oxygen species (ROS) in fruits, which can initiate lipid peroxidation and cause oxidative damage to cell membrane, chloroplasts, mitochondria and apoplast. Plants have the most effective non-enzymatic (phenolics, flavonoids, carotenoids, ascorbic acid, tocopherols, glutathione and proline) and enzymatic (catalase (CAT), superoxide dismutase (SOD), ascorbate peroxidase (APX), glutathione reductase (GR), monodehydroascorbate reductase (MDHAR) and dehydroascorbate reductase (DHAR)) antioxidant defense systems to protect cells against oxidative stress (*5*). Thus, for the improvement of antioxidant capacity and the maintenance of the quality of plum fruits during the postharvest life, suitable postharvest technologies should be considered in combination with cold storage.

Recent reports have shown that the administration of some treatments, such as salicylic acid (*6*) and oxalic acid application (*7*), hot water dipping (*8*) and nitric oxide fumigation (*9*) increase the plum quality for longer periods than cold storage alone. Phenylalanine is an aromatic amino acid used for the biosynthesis of all phenolic compounds *via* the phenylpropanoid pathway. In this pathway, phenylalanine ammonia lyase (PAL) is the first enzyme that catalyzes the conversion of phenylalanine to ﬂavonoids, phenolics and anthocyanins. Various biotic and abiotic stresses may stimulate PAL activity, which results in the accumulation of bioactive compounds (*10*). Recently, the application of phenylalanine solution as preharvest and postharvest fruit treatments has been considered for enhancing the nutritional quality of horticultural crops. Preharvest spraying with phenylalanine improved the content of phenolic compounds, including anthocyanins and flavonoids, in grape fruits (*11*). Aghdam *et al*. (*10*) reported that phenylalanine application significantly decreased chilling injury, membrane lipid peroxidation and ROS accumulation in tomato fruits during cold storage. Moreover, in fruits treated with 0.5 mM phenylalanine, the contents of phenols and flavonoids and the PAL activity were higher than in those treated with other treatments, including control fruits. Phenylalanine-treated fruits also showed higher activities of antioxidant enzymes SOD, CAT, APX and GR, concurrent with a higher endogenous accumulation of lycopene and proline. Kumar Patel *et al*. (*12*) showed that the preharvest or postharvest treatment with phenylalanine of mango, avocado, strawberry and citrus fruits activates their natural defense responses and thus tolerance to fungal pathogens, leading to the inhibition of postharvest decay caused by different fungal pathogens. However, few reports have shown the positive potential of phenylalanine in the reduction of chilling injury and maintenance of postharvest quality in horticultural crops. Therefore, the main purpose of the present study is to investigate the influence of the exogenous application of phenylalanine on fruit quality, chilling tolerance and antioxidant capacity of ‘Stanley’ plums during 40 days of cold storage.

## MATERIALS AND METHODS

### Chemicals

The chemicals used in this study were of analytical grade. Phenylalanine, trichloroacetic acid, 2,6-dichloroindophenol, metaphosphoric acid, Folin–Ciocalteu reagent, sulfosalicylic acid, ninhydrin, proline, l-methionine, potassium iodide, riboflavin and phosphate buffer were from Merck, Darmstadt, Germany. Thiobarbituric acid, 2,2-diphenyl-1-picrylhydrazyl, gallic acid, nitroblue tetrazolium, glacial acetic acid, EDTA, bovine serum albumin, polyvinylpyrrolidone (PVP), sodium carbonate and methanol were from Sigma-Aldrich, Merck, St. Louis, MO, USA.

### Fruits, treatments and storage

‘Stanley’ plum fruits were hand-picked at the commercial maturity stage (Brix° 12.01) from a commercial orchard in Zanjan province, Iran. Fruits were immediately transferred to the postharvest lab and graded based on the size and the absence/presence of defects. They were rinsed gently with water, dried naturally at room temperature, and randomly divided into four groups: control (distilled water) and phenylalanine (C_9_H_11_N_1_O_2_) treatments (2.5, 5 or 7.5 mM) for 10 min at 25 °C. Fruits were then dried at room temperature for about 30 min and placed in open plastic boxes, each containing 10 plum fruits (120 fruits for each treatment). Then, all fruits were stored at 1 °C and (90±2) % RH for 40 days. Following 10, 20, 30 and 40 days of cold storage, three boxes (three replications of each treatment) for each treatment were transferred to the chamber with a controlled temperature and maintained at 25 °C for one day. A total of approx. 40 g fruit tissue was collected from 5 fruits per replication of each treatment, frozen at once in liquid nitrogen, kept at -80 °C for later biochemical measurements.

### Fruit quality (mass loss, firmness, soluble solid content)

The fruit mass of each sample (box) was measured at the beginning of the storage and at each sampling time. The results were expressed as the percentage loss of the initial mass. Fruit firmness was evaluated in kPa using a texture analyzer (FT011; Facchini srl, Alfonsine (Ra), Italy), fitted with an 8-mm diameter flat probe. A portable refractometer (PAL-1; Atago Co., Tokyo, Japan) was used to evaluate the soluble solid content of plum juice in °Brix.

### Chilling injury and membrane integrity

The chilling injury (CI) index was determined after the fruits were kept at 25 °C for one day. The CI symptoms were gel breakdown, flesh woolliness, and flesh browning (*13*), which were assessed visually according to the following five-stage scale: 0=none, 1=slight injury, 2=moderate injury, 3=moderately severe injury and 4=severe injury. The chilling injury index was calculated based on the following formula:

CI index=∑ [(CI_scale_)·(*N*(fruit)_CIscale_)]/(4·*N*(fruit)_treated total_) /1/

Electrolyte leakage (EL) was assayed using the method of Promyou *et al.* (*14*) and calculated using the following equation:

EL=(Initial electrolyte leakage/Final electrolyte leakage)·100 /2/

The molality of malondialdehyde (MDA) was determined using the procedure developed by Dhindsa *et al.* (*15*) with some modifications. One gram of fruit samples was crushed in 10 mL of 5% (*m*/*V*) trichloroacetic acid (TCA) and then centrifuged (Z36HK; Hermle Labortechnik GmbH, Wehingen, Germany) at 10 000×*g* for 15 min. The supernatant (2 mL) was mixed with 2 mL of 5% TCA containing 0.5% thiobarbituric acid. The reaction solution was heated in a boiling water bath at 100 °C for 30 min. The mixture was cooled quickly and finally centrifuged at 10 000×*g* for 10 min. The absorbance was read at 532 and 600 nm using a spectrophotometer (Specord 250; Analytik Jena AG, Jena, Germany). Results were expressed in μmol/kg.

### Fruit biochemical factors (total phenolic, favonoid and anthocyanin contents, ascorbic acid, total antioxidant activity, phenylalanine ammonia lyase and polyphenol oxidase enzyme activity)

For the determination of total phenolic, total flavonoid and total anthocyanin mass fractions, fruit samples (1 g) were extracted with 0.1% HCl-acidified 80% methanol (10 mL) and then centrifuged (12 000×*g*, 20 min, 4 °C). Total phenolic value of the extracts was assayed with Folin–Ciocalteu reagent method (*16*), evaluated against a gallic acid standard curve and finally expressed in mg gallic acid (GAE) per 100 g fresh mass. Total flavonoid content of the extracts was evaluated following Bouayed *et al*. (*17*) method with the absorbance read at 510 nm and expressed in mg catechin per 100 g fresh mass. Total anthocyanin levels were determined following the pH differential method (*18*) and a molar absorption coefficient of 29 600 (cyanidin 3-glucoside) and expressed in mg cyanidin-3-glucoside per 100 g fresh mass according to the equation below:

*A*=(*A*_520 nm at pH=1_−*A*_700 nm at pH=1_)−(*A*_520 nm at pH=4.5_−*A*_700 nm at pH=4.5_) /3/

The level of ascorbic acid was measured following 2,6-dichloroindophenol titrimetric method (*19*). A mass of 10 g of fruit samples was mixed uniformly in 3% (*V*/*V*) metaphosphoric acid and filtered through two layers of cheesecloth. The supernatant (10 mL) was titrated against the standard 2,6-dichloroindophenol dye until the faint pink colour persisted for 5 s and the result was expressed in mg ascorbic acid per 100 g fresh mass. The antioxidant activities of the methanolic extract of the fruit samples were estimated using 2,2-diphenyl-1-picrylhydrazyl (DPPH) free radical scavenging assay (*20*). An aliquot (0.1 mL) of the extract was added to 1.9 mL of a 0.1 mM methanolic solution of DPPH and incubated for 30 min at room temperature in the darkness. After incubation, the absorbance was read against a blank at 517 nm using a spectrophotometer (Specord 250; Analytik Jena AG). The percentage of the inhibition of the DPPH radical was calculated by the following formula:

DPPH inhibition=[(*A*_control_−*A*_sample_)/*A*_control_]·100 /4/

where *A*_control_ is the absorbance of the DPPH solution without the extract.

Phenylalanine ammonia lyase (PAL) activity was assayed following the procedure described by Nguyen *et al*. (*21*) and expressed in katals produced per mass of protein (kat/kg). The activity of polyphenol oxidase (PPO) was assessed following the method described by Kahn (*22*). For this purpose, the increase in absorbance of 1.5 mL of the reaction mixture, including citrate (100 mM), phosphate buffer (200 mM, pH=5), catechol (0.05 M), and the supernatant (100 μL), was measured at 420 nm for 2 min. The activity of PPO was expressed in kat/kg.

### Proline determination

For the determination of proline, 1 g fruit tissue was uniformly mixed in 10 mL sulfosalicylic acid (3%) and subsequently centrifuged (10 000×*g*, 15 min). The obtained supernatant (2 mL) reacted with ninhydrin (2 mL, 2.5%) and glacial acetic acid (2 mL), was heated at 100 °C for 60 min, and then cooled down in an ice bath. Each tube received toluene (4 mL) and was later shaken dynamically until it was separated into two phases. The absorbance of the proline-containing phase was measured at 520 nm, and proline value (µg/g) on fresh mass basis was calculated with standard curve of known concentrations of proline (*23*).

### Extractions and assays of antioxidant enzyme activities and H_2_O_2_ content

Frozen plum fruits (1 g) were ground and extracted for 30 s with phosphate buffer (50 mM, pH=7.8) containing EDTA (0.2 mM) and PVP (2%). The homogenates were centrifuged (12 000×*g*, 4 °C, 20 min) to obtain the supernatants for enzymatic assays. For the catalase (CAT) assay, H_2_O_2_ (15 mM) and phosphate buffer (pH=7) were added to 0.1 mL extract, and the absorbance was recorded at 240 nm for 60 s. For the ascorbate peroxidase (APX), the reaction mixture (2 mL) consisted of 20 μL supernatant, phosphate buffer (pH=7), ascorbic acid (0.5 mM) and H_2_O_2_ (1 mM). The absorbance of the reaction mixture was recorded at 290 nm for 60 s. To measure the activity of superoxide dismutase (SOD), the supernatant (50 μL), phosphate buffer (25 mM, pH=7), l-methionine (12 mM), nitroblue tetrazolium (NBT; 1 M), riboflavin (1 M) and sodium carbonate (50 mM, pH=10.2) were mixed to reach a 3-mL reaction mixture. Then, the absorbance was measured at 560 nm after exposure to light for 30 min, demonstrating the enzyme ability to inhibit photochemical reduction of the NBT. The only difference from the blank mixture was the exposure to the dark for 30 min (*24*). The protein content was determined following Bradford’s (*25*) method using bovine serum albumin (BSA) as the standard. For the measurement of H_2_O_2_, 1 g of fruit tissue was homogenized in TCA (5 mL, 1% *V*/*V*) and centrifuged (10 000×*g*, 5 min). After that, 750 µL phosphate buffer (100 mM) and 1.5 mL potassium iodide (1 M) were added to the extract, the absorbance of the reaction mixture was measured at 390 nm (*26*) and results were expressed as molality in μmol/kg.

### Experimental design and data analysis

The experiments were performed in a completely randomized design. The statistical analysis was carried out using SPSS software v. 20.0 (*27*). Experimental data were subjected to ANOVA analysis. The treatments and storage were sources of variation. All treatments were conducted with three replications, and each replication contained 10 fruits. Mean values were compared using Duncan’s multiple range test (p≤0.05).

## RESULTS AND DISCUSSION

### Plum fruit quality

The mass loss of plums increased during cold storage, which was higher in control fruits than those treated with phenylalanine, while there were no significant differences among the phenylalanine-treated fruits. During 40 days of storage, control fruits lost approx. 6.01% of their mass, while those treated with phenylalanine lost 4.20% (p<0.05). During storage, the firmness of all fruits decreased significantly, but the control fruits had a faster softening rate (Table 1). It seems that the application of phenylalanine could retard tissue softening but significant differences were not observed among the phenylalanine-treated fruits. Total soluble solids, as a maturity and quality parameter, and harvest index are very important measurements for plum fruits. The soluble solid content was 12.01% at harvest and increased to 15% during 40 days of storage at 1 °C, but there were no significant differences between the experimental groups. Fruit mass loss is an important factor affected by low temperatures during cold storage. The main reason for the mass loss of fruits is the water loss due to physiological activities such as transpiration and respiration (*28*). The harvest time and maturity stage of plums directly affect their firmness (*29*). Fruit softening during storage significantly reduces the postharvest life and increases the susceptibility to fungal infection. In this investigation, the application of phenylalanine effectively delayed the softening of plum fruits, thus helping to maintain their quality. During fruit ripening and postharvest life, the mechanical strength of the cell walls and cell to cell stickiness reduces and so does the firmness of the fruits (*30*). Furthermore, Garde-Cerdán *et al.* (*31*) reported that the preharvest application of compounds containing nitrogen such as proline and phenylalanine increased amino acid concentrations and fruit quality of ‘Tempranillo’ grapes. They reported that the foliar application of phenylalanine increased the synthesis of fermentative volatile compounds, thereby improving wine quality. Seemingly, phenylalanine treatment could be an efficient method to increase plum fruit quality during cold storage.

**Table 1:** Mass loss and firmness in control samples or plums treated with phenylalanine at different concentrations. Fruits were stored at 1 °C for up to 40 days

*t*(storage)/day	*c*(phenylalanine)/mM	mass loss/%	firmness/kPa
0	Control 2.5 5 7.5	- - - -	(282.4±3.9)^a^ (282.4±3.9)^a^ (282.4±3.9)^a^ (282.4±3.9)^a^
10	Control 2.5 5 7.5	(1.2±0.06)^ef^ (1.1±0.1)^efg^ (1.5±0.2)^ef^ (0.5±0.1)^fg^	(169.6±7.8)^de^ (182.4±2.9)^cde^ (254.9±9.8)^ab^ (294.2±9.8)^a^
20	Control 2.5 5 7.5	(3.6±0.2)^bc^ (2.6±0.4)^cd^ (1.9±0.1)^de^ (2.0±0.2)^de^	(118.6±2.9)^fgh^ (137.3±2.9)^efg^ (196.1±19.6)^cd^ (245.1±9.8)^ab^
30	Control 2.5 5 7.5	(4.6±0.19)^b^ (4.0±0.2)^b^ (3.8±0.1)^b^ (3.5±0.1)^bc^	(92.2±3.9)^gh^ (119.6±2.9)^fgh^ (152.9±13.7)^def^ (225.5±9.8)^bc^
40	Control 2.5 5 7.5	(6.0±0.3)^a^ (4.3±0.2)^b^ (4.5±0.4)^b^ (4.0±0.3)^b^	(12.7±0.9)^I^ (83.3±9.8)^h^ (98.0±19.6)^gh^ (149.0±7.8)^def^
Significant	Df		
Time	4	**	**
Treatment	3	**	**
Time × Treatment	12	ns	**

Data are presented as mean value±S.D. of three replications. Different letters indicate significant differences among treatments according to Duncan’s test at p=0.05. * and ** significant differences at p<0.01 and p<0.05, respectively. Df=degree of freedom, ns=not significant

### Plum fruit chilling injury and membrane integrity

**Fig. 1:**
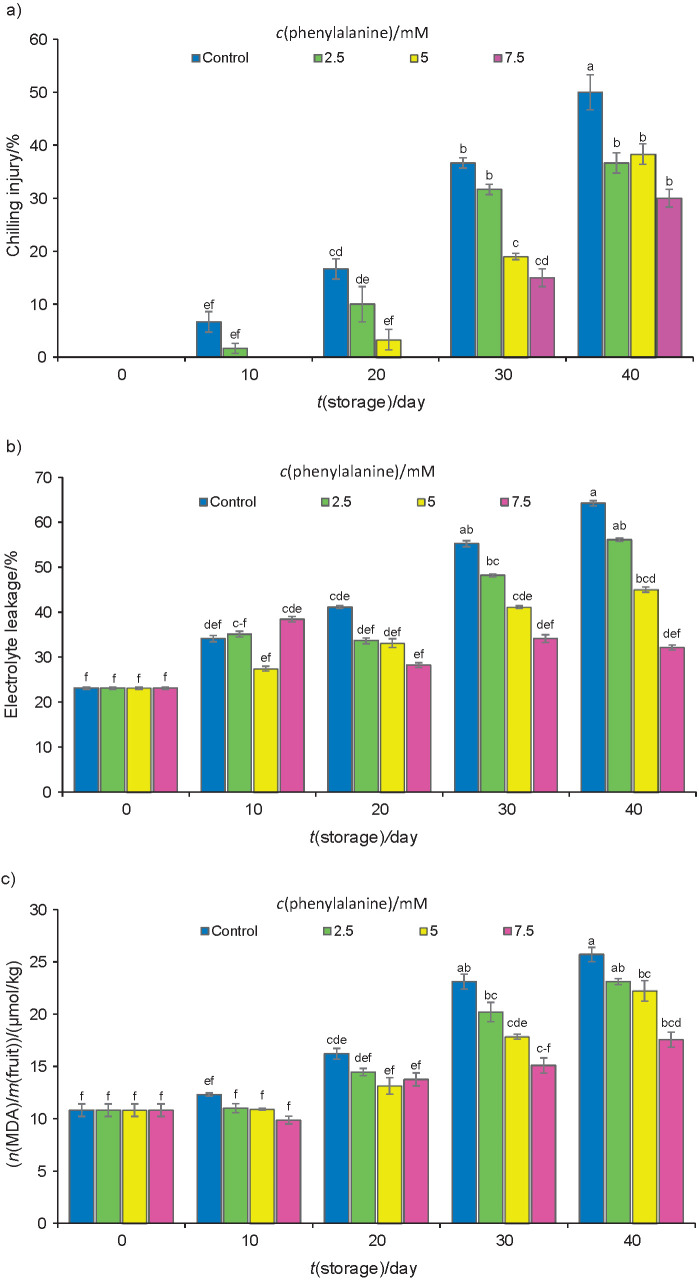
The rate of cold damage of plums (control and treated with different concentrations of phenylalanine: a) chilling injury index, b) electrolyte leakage, and c) molality of malondialdehyde (MDA) on fruit fresh mass basis. Fruits were stored at 1 °C for up to 40 days. Data are presented as mean value±S.D. of three replications. Different letters indicate significant differences among treatments according to Duncan’s test at p=0.05

For the first time, the control fruits and those treated with 2.5 mM phenylalanine showed translucency and internal browning at 1 °C after ten days of storage. The rate of cold damage increased in all fruits as time passed (Fig. 1a). However, phenylalanine treatments of 5 and 7.5 mM decreased the chilling index throughout storage and subsequently increased shelf-life period. Meanwhile, during storage for 40 days at 1 °C, the plums were chilled, the cell membrane was damaged, and so the electrolyte leakage and MDA accumulation increased. The ion leakage and the MDA concentration significantly increased with the passage of time in all fruits (Fig. 1b and Fig. 1c). However, the application of a 7.5 mM solution of phenylalanine reduced their rates, but there was no significant difference among phenylalanine treatments regarding electrolyte leakage and MDA concentration. In this study, chilling injury had a positive correlation with electrolyte leakage and MDA content. However, negative correlations were observed between the chilling injury and ascorbic acid mass fraction and antioxidant capacity (Table 2). The optimal method for the storage of stone fruits is cold storage, but plums are susceptible to cold and might exhibit chilling injury disorder (*32*). Thus, with the development of chilling symptoms, the storage life is limited. However, cultivar, storage temperature, maturity stage, and cultivation conditions can affect the rate of chilling injury development in plums (*3*). The chilling injury increased after ten days of cold storage at 1 °C plus one day at 25 °C (Fig. 1a). Candan *et al.* (*4*), working on ’Larry Ann’ plums, have already reported similar results. Moreover, the measurements of electrolyte leakage and MDA accumulation in cold-stressed plants are practical methods for assessing membrane integrity (*33*). Electrolyte leakage is a reliable indicator of cell membrane damage and is widely used for the assessment of fruit chilling injury (*34*). Peroxidation of lipids at low temperatures can modify cell membrane structure. During the cold stress, cell membrane peroxidation occurred, and MDA was produced, which damaged the fruit cell membranes. The existence of both saturated and unsaturated fatty acids in cell membranes might reduce ion leakage and MDA accumulation, thereby preventing membrane lipid peroxidation and cell damage. Furthermore, the accumulation of reactive oxygen species (ROS) and the activities of cell wall destructive enzymes damage cell membranes (*6*). Electrolyte leakage and MDA concentration in control and treated fruits were increasing during cold storage (Fig. 1b and Fig. 1c), but phenylalanine treatment at concentration of 7.5 mM reduced the harmful effects of chilling injury by preventing the peroxidation of membrane lipids. Additionally, the application of 7.5 mM phenylalanine increased the resistance of tissue to chilling injury and maintained the integrity of the cell membrane, in comparison with control fruits. These were due to the capacity of phenylalanine to increase the activity of antioxidant enzymes and the accumulation of ROS scavenging agents, such as ascorbic acid (AA), phenols, flavonoids and proline. Our results are similar to those of phenylalanine-treated tomatoes, which showed an increase in the membrane integrity and resistance of tissues to chilling injury during storage (*10*). Regarding our results, MDA concentrations were lower in phenylalanine-treated fruits, and therefore they better maintained the integrity of the membrane. This is in agreement with the results from a previous study on the exogenous application of phenylalanine on tomatoes during cold storage (*10*). The increased ROS scavenging activity following phenylalanine application might be a possible positive response of plum fruits to low temperature stress during cold storage. Kusvuran *et al.* (*35*) reported that fruit chilling injury was due to oxidative stress from ROS accumulation, which further damaged cell membrane. Furthermore, Blokhina *et al.* (*36*) have revealed that plant tissues that have better antioxidant systems better resist low temperatures. Nukuntornprakit *et al.* (*37*) reported that the development of chilling injury symptoms was correlated with ROS metabolism, so that it might reduce total antioxidant capacity. The result indicates that the high antioxidative potential of the fruits treated with 7.5 mM phenylalanine is responsible for the low chilling injury.

**Table 2:** Pearson's simple correlation coefficient among chilling injury (CI), total flavonoids (TF), total phenols (TP), ascorbic acid (AA), antioxidant capacity (AC), phenylalanine ammonia lyase (PAL), ascorbate peroxidase (APX), catalase (CAT), superoxide dismutase (SOD), polyphenol oxidase (PPO), electrolyte leakage (EL), malondialdehyde (MDA) and hydrogen peroxide (H_2_O_2_) of the phenylalanine-treated plum fruits

H_2_O_2_	MDA	EL	PPO	SOD	CAT	APX	PAL	AC	AA	TF	TP	CI	
0.64**	0.80**	0.68**	0.84**	0.47**	0.43**	0.55**	0.64**	-0.41**	-0.77**	0.34**	0.26*	1	CI
-0.19 ^ns^	0.07 ^ns^	-0.07 ^ns^	0.06 ^ns^	0.70**	0.72**	0.65**	0.73**	0.50**	0.10 ^ns^	0.85**	1	0.26*	TP
-0.09 ^ns^	0.17 ^ns^	-0.03 ^ns^	0.16 ^ns^	0.77**	0.76**	0.68**	0.74**	0.43**	0.09 ^ns^	1	0.85**	0.34**	TF
-0.80**	-0.79**	-0.77**	-0.87**	-0.22 ^ns^	-0.07 ^ns^	-0.39**	-0.39**	0.45**	1	0.09 ^ns^	0.10 ^ns^	-0.77**	AA
-0.40**	-0.40**	-0.39**	-0.40**	0.38**	0.36**	0.27*	0.24 ^ns^	1	0.45*	0.43*	0.50*	-0.41*	AC
0.29*	0.44**	0.36**	0.51**	0.81**	0.76**	0.88**	1	0.24	-0.39**	0.74**	0.73**	0.64**	PAL
0.27*	0.36 ^ns^	0.37**	0.46**	0.81**	0.78**	1	0.88**	0.27*	-0.39**	0.68**	0.65**	0.55**	APX
0.05^ns^	0.18 ^ns^	0.11 ^ns^	0.23 ^ns^	0.76**	1	0.78**	0.76**	0.36**	-0.07 ^ns^	0.76**	0.72**	0.43**	CAT
0.10^ns^	0.29*	0.24^ns^	0.31*	1	0.76**	0.81**	0.81**	0.38**	-0.22 ^ns^	0.77**	0.70**	0.47**	SOD
0.74**	0.79**	0.70**	1	0.31*	0.23 ^ns^	0.46**	0.51**	-0.40**	-0.87**	0.16 ^ns^	0.06 ^ns^	0.84**	PPO
0.68**	0.75**	1	0.70**	0.24^ns^	0.11 ^ns^	0.37**	0.36**	-0.39**	-0.77**	-0.03 ^ns^	-0.07 ^ns^	0.68**	EL
0.66**	1	0.75**	0.79**	0.29*	0.18 ^ns^	0.36**	0.44**	-0.40**	-0.79**	0.17 ^ns^	0.07 ^ns^	0.80**	MDA
1	0.66**	0.68**	0.74**	0.10^ns^	0.05 ^ns^	0.27*	0.29*	-0.40**	-0.80**	-0.09 ^ns^	-0.19 ^ns^	0.64**	H_2_O_2_

* and ** significant differences at p<0.01 and p<0.05, respectively, ^ns^=not significant respectively.

### Biochemical factors

**Fig. 2:**
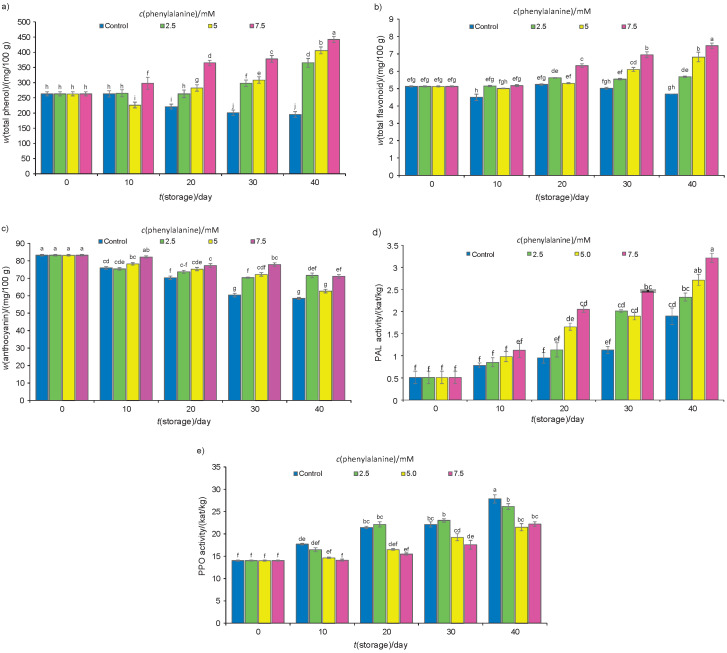
Determination on fruit fresh mass basis of: a) total phenols as gallic acid equivalents, b) total flavonoids as catechin equivalents, c) anthocyanins as cyanidin-3-glucoside equivalents and d) phenylalanine ammonia lyase (PAL) activity on protein basis,and e) PPO activity in kat/kg protein. Fruits were stored at 1 °C for up to 40 days. Data are presented as mean value±S.D. of three replications. Different letters indicate significant differences among treatments according to Duncan’s test at p=0.05

Total phenol content at harvest, expressed as gallic acid equivalents on fresh mass basis, was 263.12 mg/100 g, which was, during storage, quickly reduced in control fruits but significantly increased in the treated fruits. After 40 days of cold storage, fruits treated with 7.5 mM phenylalanine (Fig. 2a) had the highest total phenol mass fraction. During storage, the mass fraction of total phenols in control fruits decreased from 263.12 to 195.37 mg/100 g but increased to 365.14, 406.24 or 442.13 mg/100 gin those treated with 2.5, 5 or 7.5 mM phenylalanine, respectively. The mass fraction of total flavonoids in fruits treated with 7.5 mM phenylalanine was higher than in those of the other treatments after 40 days of storage (Fig. 2b). At the beginning of the experiment and after 40 days of storage, total anthocyanin mass fraction expressed as cyanidin 3-glucoside on fresh mass basis was 83.20 and 58.46 mg/100 g in control fruits, respectively. Total anthocyanin mass fraction decreased more in control than in the treated fruits (Fig. 2c). After 40 days of storage, fruits treated with 7.5 mM phenylalanine had a higher total anthocyanin mass fraction than the other experimental groups. During 40 days of storage at 1 °C, the mass fraction of ascorbic acid also decreased in all fruits. However, phenylalanine treatments maintained a higher ascorbic acid mass fraction during storage (Table 3). Finally, the mass fraction of ascorbic acid on fresh mass basis decreased to 16.67, 18.75, 29.17 and 31.67 mg/100 g in control and fruits treated with 2.5, 5 and 7.5 mM phenylalanine, respectively. Before the treatments, antioxidant activity was 79% based on the fresh mass, which gradually declined in control fruits during the experiment. The antioxidant activity in the treated fruits increased during the first 20 days of storage but then it decreased. After 40 days of storage, the antioxidant activity in fruits treated with 7.5 mM phenylalanine was greater than in those of the other treatments (Table 3). As shown in Fig. 2d, the activity of PAL in all fruit samples enhanced during storage. However, during storage, PAL activity in the control fruits was lower than in the treated ones. After 40 days of storage, the activity of PAL in the fruits treated with 7.5 mM phenylalanine was higher than in the other fruits (Fig. 2d). Our results showed that the activity of PPO increased during cold storage (Fig. 2e). The activity of PPO in fruits treated with 7.5 mM phenylalanine was significantly lower than in those of the other treatments. On the first day, the activity of PPO on protein basis was 14.01 kat/kg and increased to 27.85 and 22.20 kat/kg in control and the fruits treated with 7.5 mM phenylalanine, respectively. The results showed that flavonoids, phenols, ascorbic acid and anthocyanins were present at remarkably higher levels in fruits treated with phenylalanine than in untreated fruits. In fruits treated with phenylalanine, a higher activity of PAL is accountable for the higher accumulation of phenolic compounds. In the current study, PAL positively correlated with total phenols, flavonoids, and antioxidant activity (Table 2). The increased PAL activity has been correlated with an increased production of phenylpropanoid (*38*). The phenylpropanoid pathway has been extensively studied concerning the production of a wide variety of natural phenolic compounds such as isoflavonoids, flavonoids, hydroxycinnamic acids, coumarins, lignin and stilbenes. In addition, PAL is an important enzyme during the biosynthesis of anthocyanins in plums, and its activities result in the accumulation of anthocyanins (*7*). Therefore, the increased PAL activity promoted the phenylpropanoid pathway (Fig. 2d) in plums in response to the exogenous phenylalanine application at 7.5 mM, which resulted in a higher accumulation of phenolic compounds (Fig. 2a-c), thereby enhancing the DPPH scavenging capacity. These biocompounds might be involved in the antioxidant capacity. It seems that the increased mass fractions of phenolic compounds and anthocyanins following the administration of phenylalanine treatments were probably responsible for the increased antioxidant capacity as measured by DPPH assays. PPO, as a basic enzyme, is responsible for the browning of cold-damaged horticultural crops. In the present experiment, during cold storage, the activity of PPO increased in all samples and was greater in control than in phenylalanine-treated fruits (Fig. 2e). Martinez and Whitaker (*39*) reported that the activity of PPO increased during storage at 0 °C, and it might be a chilling injury symptom. It was suggested that the increased PPO activity was in response to the chilling of plum fruits, which implicated a role for PPO in the development of flesh browning. In this study, the activity of PPO had a positive correlation with the chilling injury of plum fruits (Table 2). Therefore, a low activity of PPO in phenylalanine-treated fruits appears to be related to a less strong occurrence of chilling injury. The membrane penetrability and the interplay of phenols and PPO, which are commonly found in separate cellular compartments, take place throughout chilling injury disturbance (*40*). A relationship between polyphenol content and the activity of PPO was achieved in the early and late harvested fruits during the postharvest period. In this research, TP reduced during storage in control plums, and their decrease was accompanied by an increase in the activity of PPO. Ascorbic acid is an anti-browning agent that acts as an inhibitor of PPO activity (*41*). The mass fraction of ascorbic acid in the fruits decreased during storage, and a supposition has been developed (*42*) which states that a higher ascorbic acid mass fraction leads to a weaker susceptibility of fruit to flesh browning. Although PPO has been strongly associated with flesh browning of fruits, other enzymes such as peroxidase (POD) and PAL have also been studied for the development of the disorder, most recently in pineapples (*43*-*45*). POD has been shown to be involved in the discolouration of numerous fruits and vegetables. However, its association with flesh browning is still questioned as both lower and higher POD activities have been reported. Zhou *et al.* (*44*) observed that chilling injury increased the activity of PAL and stimulated the biosynthesis of polyphenol compounds in pineapple. Furthermore, there was no direct correlation between the PAL activity and flesh browning development (*45*).

**Table 3:** Ascorbic acid (AA) mass fraction on fruit fresh mass basis and antioxidant activity expressed as DPPH inhibition in control group or plums treated with phenylalanine at different concentrations. Fruits were stored at 1 °C for up to 40 days

*t*(storage)/day	*c*(phenylalanine)/mM	*w*(AA)/(mg/100 g)	Inhibition of DPPH radical/%
0	Control 2.5 5 7.5	(39.2±0.5)^a^ (39.2±0.5)^a^ (39.2±0.5)^a^ (39.2±0.5)^a^	(79.0±0.2)^def^ (79.0±0.2)^def^ (79.0±0.2)^def^ (79.0±0.2)^def^
10	Control 2.5 5 7.5	(30.0±0.8)^def^ (35.0±0.01)^bc^ (34.2±0.9)^bc^ (35.8±0.9)^ab^	(78.9±0.2)^def^ (80.0±0.2)^b-f^ (79.8±0.3)^b-f^ (81.1±0.5)^a-d^
20	Control 2.5 5 7.5	(23.3±0.5)^gh^ (26.7±0.5)^fg^ (34.7±0.5)^bc^ (36.7±1.3)^ab^	(78.4±0.65)^f^ (80.5±0.32)^b-f^ (81.4±0.55)^abc^ (82.8±0.62)^a^
30	Control 2.5 5 7.5	(23.0±1.1)^gh^ (22.0±0.7)^h^ (26.7±0.5)^fg^ (33.2±0.6)^bcd^	(75.6±0.4)^g^ (79.5±0.3)^c-f^ (80.9±0.4)^a-e^ (82.0±0.5)^ab^
40	Control 2.5 5 7.5	(16.7±0.5)^j^ (18.7±0.7)^ij^ (29.2±1.3)^ef^ (31.7±0.5)^cde^	(75.0±0.4)^g^ (78.7±0.4)^ef^ (79.3±0.4)^c-f^ (80.1±0.3)^b-f^
Significant	Df		
Time	4	**	**
Treatment	3	**	**
Time × Treatment	12	**	**

Data are presented as mean value±S.D.of three replications. Different letters indicate significant differences among treatments according to Duncan’s test at p=0.05. * and ** significant differences at p<0.01 and p<0.05, respectively. Df=degrees of freedom, ns=not significant

### Proline mass fraction

**Fig. 3:**
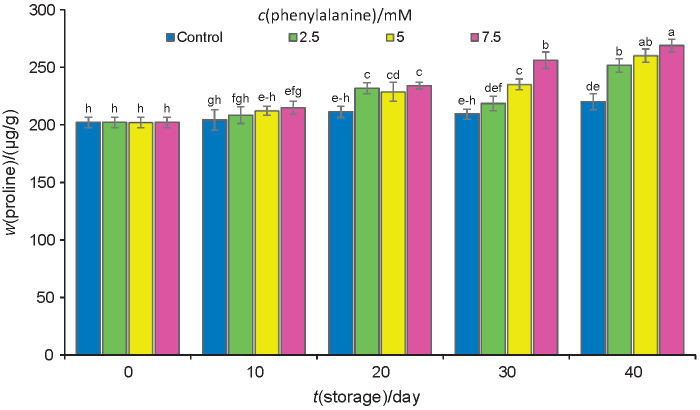
Proline mass fraction on fresh mass basis in control samples and plums treated with phenylalanine at different concentrations. Fruit were stored at 1 °C for up to 40 days. Data are presented as mean value±S.D. of three replications. Different letters indicate significant differences among treatments according to Duncan’s test at p=0.05

Proline mass fraction increased in fruits during storage at 1 °C for 40 days (Fig. 3). The exogenous application of phenylalanine resulted in a statistically superior proline accumulation during storage in the fruits treated with phenylalanine. After 40 days of storage, the proline accumulation in the fruits treated with 7.5 mM phenylalanine was higher than in those treated with 2.5 or 5 mM phenylalanine (Fig. 3). The accumulation of proline, as an osmoregulator, alleviated the stress in the fruits. By inhibiting enzymatic deterioration and scavenging hydroxyl radicals *via* osmoregulation, proline enhanced the endurance of plants against stress (*46*), thus protecting membrane integrity and antioxidant enzymes. The accumulation of proline could improve cold tolerance in cold-sensitive plants (*47*). Our results are in line with the previous findings that showed that phenylalanine treatment increased proline content (*10*), which led to an increased cold tolerance in plums.

### Activities of antioxidant enzymes and H_2_O_2_ concentration

**Fig. 4:**
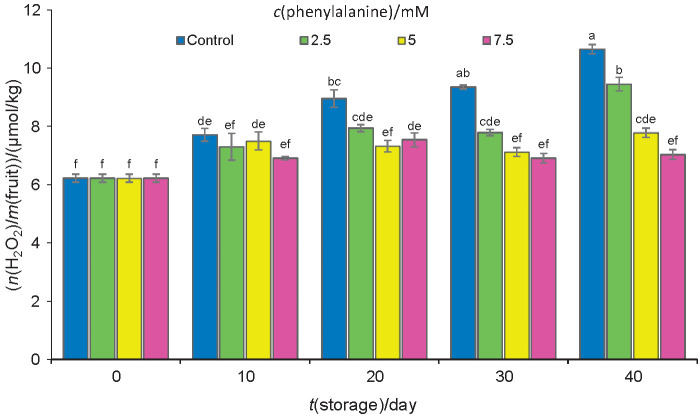
Molality of H_2_O_2_ on fresh mass basis in control samples and plums treated with phenylalanine at different concentrations. Fruits were stored at 1 °C for up to 40 days. Data are presented as mean value±S.D. of three replications. Different letters indicate significant differences among treatments according to Duncan’s test at p=0.05

The activities of antioxidant enzymes (SOD, APX and CAT) in all fruits increased permanently during storage. During storage the control fruits showed relatively lowered enzyme activities in comparison with the treated fruits. At the end of the storage, the activities of SOD, APX and CAT in control fruits on protein basis were 2.35, 6.58 and 2.12 kat/kg, respectively, while they were 3.87, 9.12 and 3.64 kat/kg in the fruits treated with 7.5 mM phenylalanine, respectively (Table 4). During storage, the concentration of H_2_O_2_ increased in control and treated fruits (Fig. 4), suggesting the intensification of peroxidation in the fruits and chilling injury development. The highest and lowest concentrations of H_2_O_2_ were detected in control and the fruits treated with 7.5 mM phenylalanine. Regarding our results, a positive correlation was found between the chilling injury and the activities of antioxidant enzymes (SOD, APX and CAT) and H_2_O_2_ concentration (Table 2). The low temperatures caused chilling injury disturbance in the fruits and increased membrane lipid oxidation, which may be linked to tissue destruction (*13*). The accumulation of ROS accelerates senescence by inducing lipid peroxidation and oxidative damage (*48*). For the removal of these reactive oxygen species, plants use enzymatic (SOD, POX, APX, CAT) and non-enzymatic (antioxidant compounds such as AA) systems (*49*). Increased activities of antioxidant enzymes are one of the protection mechanisms in plants against harmful effects of ROS produced in several tissues at low temperatures (*5*). SOD is known as the first antioxidant enzyme that acts at the front line of the defense system. Several investigations have shown that CAT may be a necessary antioxidant enzyme in the chilling tolerance of susceptible fruits during low temperature storage (*50*). APX converts H_2_O_2_ into water using two molecules of AA as a reducing power with a joint production of two units of monodehydroascorbate. The increased activity of APX, utilizing AA as a substrate in the oxidation reaction to inhibit the accumulation of H_2_O_2_, has been found to be a mechanism of chilling tolerance in some horticultural crops (*3*, *51*). Therefore, the alterations in the antioxidant ingredients in plums during cold storage seem to be more significant in the protection against the oxidative damage than in chilling injury. It appears that the chilling tolerance in phenylalanine-treated fruits is due to the enhanced activities of SOD, CAT and APX. In this research, the enhanced activities of these enzymes improved the tissue capacity to detoxify ROS, leading to a delay in ripening and senescence processes. Aghdam *et al.* (*10*) reported that tomato fruits, a cold-susceptible crop, treated with phenylalanine exhibited higher activities of antioxidant enzymes such as SOD, CAT, APX and GR, which led to a higher tolerance to chilling. In this study, the application of phenylalanine significantly reduced hydrogen peroxide (H_2_O_2_) in plum fruits. Therefore, it is safe to say that treatment with 7.5 mM phenylalanine has prevented membrane lipid peroxidation in plums during storage and thus reduced chilling injury. The chilling injury may lead to the formation of reactive oxygen species such as H_2_O_2_, which harms the membrane *via* lipid peroxidation (*52*). One of the most probable reasons for the decreased H_2_O_2_ concentration in phenylalanine-treated plums during storage is the increased activities of the antioxidant enzymes SOD, CAT and APX, as they are liable for the elimination of a lot of H_2_O_2_. This research suggests that phenylalanine-treated plum fruits tolerant to low-temperature stress are equipped with a more effective antioxidative system.

**Table 4:** Catalase (CAT), ascorbate peroxidase (APX) and superoxide dismutase (SOD) activity on protein basis in control samples or plums treated with phenylalanine at different concentrations. Fruits were stored at 1 °C for up to 40 days

*t*(storage)/day	*c*(phenylalanine)/mM	CAT	Enzyme activity/(kat/kg) APX	SOD
10	Control 2.5 5 7.5	(1.7±0.1)^h^ (2.3±0.1)^def^ (2.1±0.07)^fgh^ (2.5±0.1)^c-^f	(4.7±0.4)^fg^ (4.5±0.2)^gh^ (5.2±0.3)^d-g^ (6.6±0.1)^cd^	(2.0±0.08)^fgh^ (1.9±0.1)^gh^ (2.3±0.2)^e-h^ (2.4±0.05)^d-g^
20	Control 2.5 5 7.5	(2.0±0.05)^fgh^ (2.1±0.1)^e-h^ (2.3±0.07)^def^ (2.6±0.08)^cdf^	(5.0±0.4)^efg^ (6.3±0.1)^cde^ (6.6±0.04)^cd^ (7.2±0.2)^bc^	(1.2±0.08)^gh^ (2.5±0.08)^d-g^ (2.8±0.1)^de^ (2.9±0.2)^cd^
30	Control 2.5 5 7.5	(2.2±0.07)^d-g^ (2.2±0.1)^d-h^ (2.7±0.1)^cd^ (3.1±0.1)^b^	(5.9±0.2)^c-f^ (6.1±0.1)^c-f^ (7.3±0.2)^bc^ (8.8±0.2)^a^	(2.4±0.1)^d-g^ (2.6±0.07)^def^ (3.6±0.05)^ab^ (4.0±0.1)^a^
40	Control 2.5 5 7.5	(2.1±0.06)^e-h^ (2.3±0.06)^def^ (2.9±0.1)^bc^ (3.6±0.07)^a^	(6.6±0.4)^cd^ (7.1±0.2)^bc^ (8.5±0.2)^ab^ (9.1±0.1)^a^	(2.3±0.2)^d-h^ (2.8±0.1)^def^ (3.4±0.1)^bc^ (3.9±0.1)^ab^
Significant	Df			
Time	3	**	**	**
Treatment	3	**	**	**
Time × Treatment	9	**	ns	*

Data are presented as mean value±S.D. of three replications. Different letters indicate significant differences among treatments according to Duncan’s test at p=0.05. * and ** significant differences at p<0.01 and p<0.05, respectively. Df=degree of freedom, ns=not significant

## CONCLUSIONS

A positive correlation was found between the chilling injury and electrolyte leakage and malondialdehyde concentration. Postharvest treatment of plums with phenylalanine decreased the chilling injury and protected cell membranes during storage. The low-temperature tolerance may be because of a lower accumulation of H_2_O_2_ as a result of the more active reactive oxygen species (ROS) removing the enzymes and antioxidant systems, along with a more endogenous accumulation of proline. In the current study, phenylalanine ammonia lyase (PAL) had a positive correlation with the antioxidant capacity. However, a negative correlation was found between ascorbic acid mass fraction and antioxidant capacity and chilling injury index. Consequently, high mass fractions of ascorbic acid and antioxidant capacity were accompanied by a low chilling injury index. Phenylalanine treatments significantly increased the activity of PAL in plum fruits during cold storage. These treatments maintained higher mass fractions of total flavonoids, phenolics and anthocyanins. Our results demonstrated that phenylalanine treatments effectively retarded or avoided tissue softening, maintained a higher ascorbic acid mass fraction and antioxidant capacity, and decreased the activity of polyphenol oxidese, which is responsible for the browning of plum fruits during storage. Accordingly, phenylalanine treatments may be effective in maintaining the quality of, reducing the severity of the chilling injury in, and extending the postharvest life of plums during cold storage.
